# 

**Published:** 1910-03-12

**Authors:** 


### GRADUATES' MEDICAL SCHOOLS.

DIARY FOR THE WEEK, MARCH 14 to MARCH 19.

**THE BROMPTON HOSPITAL FOR CONSUMPTION AND DISEASES OF THE CHEST, Fulham Road, S.W.**

**At 4 p.m.—March 16,** Dr. Bosanquet, **Pleurisy.**

**ST. JOHN'S HOSPITAL FOR DISEASES OF THE SKIN, Leicester Square, W.C.**

**At 6 p.m.—March 17,** Chesterfield Lecture: **The Treatment of Skin Diseases.**

**LONDON SCHOOL OF CLINICAL MEDICINE, Seamen's Hospital, Greenwich, S.E.**

**At 2.15 p.m.—March 15,** Dr. Russell Wells, **Diseases of the Thyroid.**
**2.30 p.m.—March 17,** Dr. Rankin, **Nerve Storms.**

**MEDICAL SOCIETY OF LONDON, Chandos Street, Cavendish Square, W.**

**At 8.30 p.m.—March 14, Discussion on Treatment of Colitis,** opened by Dr. H. P. Hawkins and Mr. H. S. Collier.

**NORTH-EAST LONDON POST-GRADUATE COLLEGE, Prince of Wales's General Hospital, Tottenham, N.**

**At 4.30 p.m.—March 15,** Dr. A. de Prenderville, **The Relative Values of Deep Light and Incomplete Narcosis. March 17,** Dr. A. E. Giles, **The Influence of Ovariotomy on Woman's Character.**

**CENTRAL LONDON THROAT AND EAR HOSPITAL, Gray's Inn Road, W.C.**

**At 3.45 p.m.—March 15,** Dr. Atkinson, **Nose.**
**3.45 p.m.—March 18,** Dr. Dan McKenzie, **Labyrinth.**

**THE POST-GRADUATE COLLEGE, West London Hospital, Hammersmith, W.**

**At 10 a.m.—March 14 and 17, Demonstration by Surgical Registrar.**
**10 a.m.—March 15, Demonstration by Medical Registrar.**
**12 noon.—March 14,** Dr. Bernstein, **Pathological Demonstration.**
**12.15 p.m.—**Dr. Grainger Stewart, **Practical Medicine.**
**5 p.m.—**Dr. Beddard, **Medicine Lecture VI.**
**5 p.m.—**Mr. Bidwell, **The Surgery of Gall Stones.**
**5 p.m.—**Mr. Etherington Smith, **Clinical Lecture.**

**MEDICAL GRADUATES' COLLEGE AND POLYCLINIC, 22 Chenies Street, W.C.**

**At 4 p.m.—March 14,** Dr. S. E. Dore, **Clinique (skin).**
**5.15 p.m.—March 14,** Dr. Purves Stewart, **Paralysis of Motor Cranial Nerves** (with lantern illustrations).
**4 p.m.—March 15,** Dr. Fred. Taylor, **Clinique (medical).**
**5.15 p.m.—March 15,** Sir Patrick Manson, K.C.M.G., **The Treatment of Chronic Dysentery.**
**4 p.m.—March 16,** Mr. Raymond Johnson, **Clinique (surgical).**
**5.15 p.m.—March 16,** Sir W. Watson Cheyne, Bart., **On Surgical Cases.**
**4 p.m. — March 17,** Mr. Jackson Clarke, **Clinique (surgical).**
**5.15 p.m.—March 17,** Dr. J. L. Bunch, **Itching Skin Eruptions.**
**4 p.m.—March 18,** Dr. St. Clair Thomson, **Clinique (ear, throat, and nose).**

**ROYAL SOCIETY OF MEDICINE, 20 Hanover Square, W.**

**At 8.30 p.m.—March 15, Pathological Section:**
*Papers.*—Dr. W. H. Willcox: "Gastric analysis." Dr. F. H. Thiele: "Observations on the pathology of pernicious anæmia." Dr. Parkes Weber: "A case of bile producing primary carcinoma of the liver."

**5 p.m.—March 17, Dermatological Section:**
*Cases.*—Dr. J. H. Sequeira: (1) Keratodermie blenorhagique. (2) (With Dr. Graham Little): Pseudoxanthoma elasticum. Dr. Graham Little: Case for diagnosis. And other cases.

**8.30 p.m.—March 18, Electro-Therapeutical Section:**
Dr. Franz M. Goedel: "The normal and pathological stomach and the intestines shown by $x$-ray examination" (with demonstrations and cinemato-Roentgenograms).

**The Institution Library.**

"Hospital Expenditure: The Commissariat." For the use of Managers. ... ... ... ... ... ... 2s. 6d

"The District Nursing Association; Hints on how to Start it." (Net, post free.) ... ... ... ... 1s 1d.

"A Legal Handbook for the use of Hospital Authorities." ... ... ... ... ... ... ... ... 2s. 6d.

Ledgers, Charts, Case-papers, and every kind of Institutional Literature.

Of all booksellers or of The Scientific Press, Limited, 23 & 29 Southampton Street, Strand, London, W.C.

### Gifts and Bequests.

Miss Frances Elizabeth Bingley, of Rotherham, Yorks, has left £100 to the Rotherham Hospital.

Mr. Thomas Howell, of Cardiff, has left £300 to the Glamorganshire and Monmouthshire Infirmary and Dispensary.

The Hon. Caroline Fanny Cavendish, of Stroud Green, N.W., formerly Extra Maid of Honour to Queen Victoria, has left £100 to the Brompton Hospital for Consumption.

The Manchester Royal Infirmary has received a sum of £1,000 from Mr. George Spielberg to endow a bed for the treatment of cancer patients. The bed is to be named the "Edward and Antonia Spielberg Bed."

Mr. John Gower Marychurch, of Park Place, Cardiff, retired shipowner, has left £1,050 to the Cardiff Infirmary, and to the Dunfermline Cottage Hospital such sum as may be needed to endow a bed to be called "The Mrs. J. G. Marychurch (*née* Duncanson) Bed." The ultimate residue of his property, which will amount to about £4,000, he left in equal shares to the Cardiff Infirmary and the University College of South Wales.

### THE QUESTION BOX.

**SPECIAL NOTICE.—Questions of interest affecting the honorary medical staff and hospital administration in all departments are invited. When any point arises we hope our readers will formulate it in a question and so co-operate to make this column of real value and interest. In this way the experience of the best-managed hospitals should be made helpful to the whole hospital world. We shall welcome the contribution of both questions and answers.**

**N.B.—The publication of an answer in this column does not necessarily preclude the publication of subsequent answers to the same question, for a question may involve points which require to be threshed out and fully discussed. Answers, if published, will be paid for at scale rates.**

### A CENTRAL STORE FOR HOSPITALS.

### By a Provincial Matron.

The idea of a great central store from whence we could obtain all hospital articles with the minimum of trouble and time expended, opens up an alluring prospect in one's mind. But surely the enormous outlay that would be needed before any one store could be organised to cope successfully with the various needs of the hospitals would raise the prices of nearly all the articles supplied.

In the matter of the commissariat department especially I do not think it would be at all wise for country hospitals at any rate, to have the goods from "stores." Where a county hospital or a cottage hospital exists there is usually much local interest and sympathy, and the tradesmen habitually serve the institution faithfully and well.

They pride themselves on "doing their best" for "our hospital," and give good return for fair prices. Such institutions are largely dependent on the townspeople for support, and it is only fair that the money given to help the hospital should circulate in the town which supports it. If the matron be experienced in purchasing, and she or her assistant give the orders personally, and if it be well known that prices and accounts are compared and checked, as they should be, a dishonest tradesman would have no chance against detection.

To lose the local interest (which would certainly be the case were all goods to be purchased from a distance) is a mistake, for all people are better for being brought into touch with the needs of others.

The existence of a well-managed hospital in the midst of a busy city is an influence for good amongst the people, much of which, however, must be lost if the local tradespeople are ignored. I think this is a strong argument against a "central stores" for food and general supplies.

### AN ALTERNATIVE TO THE CENTRAL STORE.

### The Suggestions of the Managing Director of a Great Stores.

It will be noticed that the men with the largest practical experience who have so far written on the subject of a great central store for hospitals are unanimous in thinking that the proposal is impracticable. A correspondent in the *Standard* some years ago, referring to a speech by the Prince of Wales, made in December 1904, suggested that a good accountant from one of the big retail business houses, where they are accustomed to analyse all sorts of expenditure, receipts, and trade, should be appointed by twelve large hospitals, with a small working staff. The accountant would give a week's work in each quarter to each of the hospitals, checking and analysing both prices and accounts. At the end of a year the accountant would present a report that should be at once intelligible and convincing; he would be able to point out exactly, almost from the start, where any given hospital was saving or losing money as against another, and in the buying department he would be in the position of the head of a big firm and able to take advantage of the lowest possible market for every article of food, clothing, medicine, and even labour. Thus everyone supplying a hospital would soon know quite well that if their prices were higher, or their goods inferior to those supplied to any other hospital, they would speedily lose their contract. The cost of putting this plan into force was not to exceed £1,500 the first year, by the end of which time the writer urged the hospitals would probably be able to save the whole of this sum between them, for they would all, having less responsibility, be able to dispense with some clerical help and to require a less experienced staff.

#### How to Reduce Prices.

The following is the extract from the speech of the Prince of Wales to which the above reference is made: "We have done all in our power to provide the necessary information by giving the comparative prices of the principal goods used in the different hospitals. Some of the discrepancies are certainly worth serious consideration. By way of example I would mention that there is a difference of no less than 3¼d. per lb. between the highest and lowest prices paid for the same description of beef, of 3½d. per lb. for legs of mutton, of 1s. 1d. in the price of fowls, of 2½d. per gallon in the price of milk, of 7d. per lb. in the price of tea. In drugs and household articles there are similar discrepancies. There may be reasons to account for some of these differences, and all we ask for is careful inquiry on the part of each hospital. I am glad to hear that in the case of one of the largest and best managed hospitals our efforts have been gratefully acknowledged, and that, as a result of the comparisons we have provided, various economies have been effected which have resulted in a considerable saving. With regard to the question of prices paid, it is obvious that a large contract has a considerable advantage over a small one. For this reason I merely venture to throw out, as a suggestion, the possibility of some of the smaller hospitals combining to make a large contract, and thus gaining the advantage enjoyed by the larger institutions. If this is feasible, and the assistance of some outside body is required to organise the preliminary arrangements, I am quite certain that our Committee would gladly give every assistance in their power by encouraging hospital managers to meet and discuss the question."

Since the Prince made his speech the saving effected in the cost of working several of the great London hospitals has been material. In 1905 a saving of no less than £21,000 on the previous year's working of 16 hospitals was effected. In 1906 the total economies at 16 hospitals represented a saving of £39,000 per annum. At the end of 1906 the cost per bed occupied in the 16 largest general hospitals had fallen from £94 10s. 9d. in 1903 to £89 7s. 8d. On January 1, 1907, a new system enabling more accurate comparison of expenditure to be made came into force, and in 1908 the average cost per bed occupied was £81 10s. 9d.

The managing director of one of the largest stores has privately expressed the opinion that he could with advantage advise on several of the chief points that would be of primary importance, in case some of the smaller hospitals combine to make a large contract, with the cooperation of the King's Fund, in the matter of the best markets to go to for supplies of all kinds. The managing director bases this belief on the fact that his stores are the largest retail buyers in the Metropolis, and so have some claim to experience in this respect, which is a very important factor where saving is one of the chief objects. The managing director affirms that the machinery by which the large business of his stores is regulated and worked is as nearly perfect in its organisation as possible, and any part of this, including the clerical section, might be placed at the disposal of those whom the King's Fund might appoint to deal with the question, all such help being, of course, in an absolutely honorary capacity. The managing director makes this offer for what it may be worth. If any useful purpose can be served, in the view of those immediately concerned, he would be only too pleased to be invited to attend a conference and give a full explanation of the scheme he has in his mind, and of the facilities he is prepared to offer for the advantage of the hospitals who may combine under some such scheme as that suggested by the Prince of Wales in his speech.

We publish these suggestions with the hope of helping the hospitals to the utmost, and should be glad to hear what view is generally taken of the practicability of these proposals, and to further the solution of a difficult question by every means in our power.

---

Dr. J. H. Honan, of Berlin, has translated and edited the official guide to Bad-Nauheim published by the Grand Ducal Administration. The guide is reliable, up-to-date, and replete with information on just those points that physicians would like to know before they send their patients to a health resort, and it is forwarded only to medical men. For a decennium the number of those visiting Bad-Nauheim in quest of health has increased to such an extent that the equipments for caring for the patients no longer suffice. The Hessian Government, which owns the springs, therefore decided to apply to its constitutional representatives for means completely to renovate and reconstruct all buildings and equipments devoted to curative purposes. In the year 1904 the grant was made and the renovation and reconstruction commenced. This work has now advanced so far that the Administration considers it advisable to call the attention of the medical profession to the state of perfection attained in connection with the methods of dispensation of its remedies. For this purpose the guide pamphlet has been prepared for the medical profession only. This contains, *inter alia*, for the first time, the results of many years of observation as to the active carbonic-acid strength of the constituents of the baths which are certain to be of interest to the medical world, as the literature in existence supplies practically no information on this subject.

### COMING EVENTS OF THE WEEK.

**Saturday, March 12.**—London Ophthalmic Exhibition. Medical Society's Rooms, 11 Chandos Street, W., 12-9. (Conferences postponed.)

Health Exhibition, Royal Agricultural Hall, Islington.

**Sunday, March 13.**—Hospital Visiting Day.

**Monday, March 14.** — Royal College of Surgeons. Professor G. Coates' Hunterian Lecture on "Congenital Abnormalities of the Eye," 5.

London School of Tropical Medicine: Dr. Daniels on "Tropical Hygiene," 15 Seething Lane, E.C., 4.

Medical Society, 11 Chandos Street, W., 8.30.

Annual court, Royal Hospital for Diseases of Chest, City Road, E.C., 3.

Health Exhibition, Royal Agricultural Hall, Islington (last day).

**Tuesday, March 15.** — Royal College of Physicians. Professor Osler's Lumleian Lecture on "Angina Pectoris," 5.

London School of Tropical Medicine: Annual dinner, Princes Restaurant, 7.30.

Discussion on Minority Report Poor Law Commission, 20 Hanover Square, W., 4.30.

**Wednesday, March 16.**—Royal Sea-Bathing Hospital, Annual meeting, 13 Charing Cross Road, 3.

**Thursday, March 17.** — London School of Tropical Medicine: Dr. Daniels on "Tropical Hygiene," 11 Seething Lane, E.C., 4.

University College Hospital, Gower Street, W.C.: Annual court, 3.30.

Royal National Hospital for Consumption: Annual meeting, 18 Buckingham Street, Strand, W.C., 3.

**Friday, March 18.**—Infants' Hospital, Vincent Square, S.W.: Annual court, 4.30.

### Appointments Vacant.

Royal Free Hospital, Gray's Inn Road.—Senior Resident Medical Officer.

University of Sheffield.—Demonstrator in Anatomy.

Seamen's Hospital Society, Greenwich.—Two House Physicians, two House Surgeons.

Royal Southern Hospital, Liverpool.—Honorary Surgeon.

Royal Albert Hospital, Devonport.—Assistant Resident Medical Officer.

---

The London County Council has resolved and ordered that Section 55 of the Public Health (London) Act, 1891, with respect to the notification of infectious disease, shall apply, in the County of London, for a further period of twelve calendar months from and including March 13, 1910, to the disease known as cerebro-spinal fever (epidemic cerebro-spinal meningitis).

